# The medicinal plant *Tabebuia impetiginosa* potently reduces pro-inflammatory cytokine responses in primary human lymphocytes

**DOI:** 10.1038/s41598-021-85211-8

**Published:** 2021-03-09

**Authors:** Rachael Y. M. Ryan, Alejandra Fernandez, Yide Wong, John J. Miles, Ian E. Cock

**Affiliations:** 1 Australian Institute of Tropical Health and Medicine (AITHM), James Cook University, Cairns, QLD 4878 Australia; 2 Centre for Molecular Therapeutics, James Cook University, Cairns, 4878 Australia; 3 School of Environment and Science, Griffith University, Brisbane, QLD 4111 Australia; 4 Centre for Tropical Bioinformatics and Molecular Biology, James Cook University, Cairns, 4878 Australia; 5 Environmental Futures Research Institute, Griffith University, Brisbane, QLD 4111 Australia

**Keywords:** Immunotherapy, Drug development, Molecular medicine

## Abstract

Bark from the *Handroanthus impetiginosus* (Mart. ex DC.) Mattos (Bignoniaceae) tree has long been used in traditional South American healing practises to treat inflammation. However, its anti-inflammatory activity has not been closely examined. Here we use chemical extraction, qualitative phytochemical examination, toxicity testing and quantitative examination of anti-inflammatory activity on human cells ex vivo. All extracts were found to be nontoxic. We found different extracts exhibited unique cytokine profiles with some extracts outperforming a positive control used in the clinic. These results verify the immunomodulatory activity of *Handroanthus impetiginosus* (Mart. ex DC.) Mattos (Bignoniaceae) tree bark-derived compounds. Collectively, combining a lack of toxicity and potency in human immune cells supports further fractionation and research.

## Introduction

Inflammation is a complex, multi-faceted response to injury, infection or dysfunctional activity against the host. Inflammation may be initiated by a variety of stimuli including burns, wounds, bites and stings and is characterised by pain, swelling redness and heat. Inflammation is a necessary process to eliminate the cause of the injury, to clear damaged cells and tissues, and to initiate tissue repair. However, inflammation may result in severe pain and discomfort, and numerous pharmaceutical interventions are aimed at blocking these effects and alleviating these symptoms. Indeed, anti-inflammatory drugs account for approximately half the clinically available analgesics^1^. Non-steroidal anti-inflammatory drugs (NSAIDs) are most used to treat acute inflammation, although steroidal anti-inflammatory drugs (SAIDs) may also be used for more severe inflammation. Unfortunately, both NSAIDs and SAIDs are toxic and induce side effects, including gastric ulcers, headaches, dizziness, high blood pressure, liver/kidney toxicity, and increases the risk of stroke and coronary disease^1,2^. There is a need to develop safer, effective anti-inflammatory therapies with a deeper understanding of inflammatory pathways.

Multiple classes of molecules coordinate to elicit an inflammatory response^2^. These cytokines and chemokines may target the cells that release them (autocrine), affect nearby cells (paracrine), or affect distant cells (endocrine/exocrine). Individual cytokines may have either pro-inflammatory or anti-inflammatory effects^3^. Blocking the secretion and down-stream effects of these molecules prevents inflammatory symptoms including pain, swelling and redness^4^. Thus, compounds that inhibit the secretion of pro-inflammatory cytokines are attractive leads for drug development. Conversely, stimulating the secretion of anti-inflammatory cytokines may have similar therapeutic effects^5^. Substantial research has focused on modulating the levels and actions of these two classes of cytokines. Whilst many of these studies have focused on the design of novel synthetic compounds and their testing, much research has also been directed to the discovery of natural anti-inflammatory compounds^6,7^. A re-examination of traditional medicines is a logical option, as in many cases, the therapeutic properties have been extensively documented, making species selection relatively simple.

*Handroanthus impetiginosus* (Mart. ex DC.) Mattos (Bignoniaceae) tree bark (also known as the Trumpet tree, Pau d’arco and Lapacho) is a large deciduous tree which native to the America’s and is particularly common in Argentina, Bolivia and Paraguay^8^. The trees are frequently found in Central and South American tropical rain forests where they reach heights of up to 40 m and are recognisable by violet-coloured flowers^8,9^. *Handroanthus impetiginosus* (Mart. ex DC.) Mattos (Bignoniaceae) tree bark has been used by indigenous South American cultures for centuries and may even pre-date the Incan civilisation. Traditionally, *Handroanthus impetiginosus* (Mart. ex DC.) Mattos (Bignoniaceae) is used as a remedy for inflammation, cancer, syphilis, malaria, fevers, trypanosomiasis, fungal infections, bacterial infections and stomach ulcers^8^.

The phytochemistry of *Handroanthus impetiginosus* (Mart. ex DC.) Mattos (Bignoniaceae) has been relatively well studied. The bark has been reported to contain substantial amounts of flavonoids^10^, cyclopentene dialdehydes^11^, as well as benzoic acid and benzaldehyde derivates^12^. However, quinones (naphthoquinones and anthraquinones) have been reported to be the most therapeutically relevant compounds in *Handroanthus impetiginosus* (Mart. ex DC.) Mattos (Bignoniaceae) bark extracts^13^. Eighteen quinones have been identified to date. The most abundant and effective compounds are the naphthoquinones lapachol and its derivative, β-lapachone^8^. Both are major contributors to anticancer activity against cancer cell lines^8^. These compounds have attracted substantial recent interest as they also have anti-inflammatory properties^14,15^.

Despite reports of *Handroanthus impetiginosus* (Mart. ex DC.) Mattos (Bignoniaceae) bark alleviating symptoms of inflammation and inhibition of some cancer cell line proliferation, the molecular mechanisms involved have not been extensively explored. Our study was undertaken to examine the effects of *Handroanthus impetiginosus* (Mart. ex DC.) Mattos (Bignoniaceae) bark extracts on human cellular and myeloid associated inflammatory responses directly ex vivo. We report for the first time that *Handroanthus impetiginosus* (Mart. ex DC.) Mattos (Bignoniaceae) bark extracts have diverse cytokine secretion profiles in primary human immune cells and are promising leads for drug development. Furthermore, we have determined the composition of the volatile phytochemicals in the extracts to help focus future studies in this field.

## Materials and methods

### Plant material and extraction

All solvents used in this study were analytical grade and obtained from Ajax Chemicals Ltd, Australia. All other reagents were obtained from Sigma Aldrich, Australia except where indicated. Coarsely ground *Handroanthus impetiginosus* (Mart. ex DC.) Mattos (Bignoniaceae) bark was supplied by Noodles Emporium (Australia) and were originally sourced from Peru. The sample was stored at − 30 °C until required. Individual 1 g masses of bark were weighed and transferred into five 50 mL Falcon tubes. Each tube was filled with 50 mL of individual solvents of varying polarity (water, methanol, ethyl acetate, chloroform and hexane). The bark was extracted for 24 h with gentle oscillation. The resultant extracts were filtered into new tubes using Whatman No. 54 filter paper. The extracts were subsequently dried at 40 °C in a vacuum incubator until the solvents had completely evaporated. The dried products were weighed and dissolved in 10 mL deionised water (containing 1% DMSO) that was syringe filtered using Millipore 0.22 µm membrane filters. Extracts were stored at 4 °C until analysis.

### Qualitative phytochemical studies

Phytochemical analysis of the *T. impetiginosa* bark extracts for the presence of alkaloids, anthraquinones, cardiac glycosides, flavonoids, phenolic compounds, phytosteroids, saponins, tannins and triterpenoids were extracted as we have previously described in detail^16^. Alkaloids were extracted using the Mayer’s reagent test and Wagner’s reagent test. Anthraquinones were extracted using the Kumar and Ajaiyeoba tests. Cardiac glycosides were extracted using the Keller–Kiliani test. Flavonoids were extracted Kumar test. Phenolic compounds were extracted using the Folin–Ciocalteu procedure. Phytosteroids were extracted using phytosterol fatty acid ester and its removal by distillation. Saponins were extracted using one ml of the pure bark extract added to 1 mL of deionised water and shaken vigorously for 30 s. The tubes remained standing for 15 min. Tannins were extracted using the ferric chloride test. Triterpenoids were extracted using the Salkowski test.

### Non-targeted GC–MS headspace analysis

As previously described^17–21^**,** separation and quantification of phytochemical components were performed using a Shimadzu GC-2010 plus (USA) linked to a Shimadzu MSTQ8040 (USA) mass selective detector system as previously described^22^. Briefly, the system was equipped with a Shimadzu auto-sampler AOC-5000 plus (USA) fitted with a solid phase micro-extraction fibre (SPME) handling system utilising a Supelco (USA) divinyl benzene/carbowax/polydimethylsiloxane (DVB/CAR/PDMS). Chromatographic separation was performed using a 5% phenyl, 95% dimethylpolysiloxane (30 m × 0.25 mm id × 0.25 µm) capillary column (Restek). Helium (99.999%) was employed as a carrier gas at a flow rate of 0.79 mL/min. The injector temperature was set at 230 °C. Sampling utilised a SPME cycle which consisted of an agitation phase at 500 rpm for a period of 5 s. The fibre was exposed to the sample for 10 min to allow for absorption and then desorbed in the injection port for 1 min at 250 °C. As previously described, the initial column temperature was held at 30 °C for 2 min, increased to 14 °C for 5 min, then increased to 270 °C over a period of 3 min and held at that temperature for the duration of the analysis. The gas chromatography-mass spectroscopy (GC–MS) interface was maintained at 200 °C with no signal acquired for 1 min after injection in split-less mode. The mass spectrometer was operated in the electron ionisation mode at 70 eV. The analytes were then recorded in total ion count (TIC) mode. The TIC was acquired after a 1 min and for a duration of 45 min utilising a mass range of 45–450 m*/z*.

### Toxicity screening

Toxicity of the extracts were tested using a modified *A. franciscana* nauplii lethality assay^23,24^. Briefly, *A. franciscana* nauplii were incubated in the presence of the extracts, reference toxin (1 mg/mL potassium dichromate) or artificial seawater (negative control) at 25 ± 1 °C under artificial light. All treatments were performed three times in triplicate (*n* = 9). Following 24 h and 48 h exposure, the number of dead nauplii were counted in each well and expressed as a percentage of the total nauplii in the well. LC_50_ was unable to be calculated.

### In vitro immune modulation and quantification

As previously described^25^, peripheral blood mononuclear cells (PBMC) were separated from venous blood of healthy volunteers 25–35 years of age that were free of reportable diseases and reported no ill health at the time of blood collection. Informed consent was obtained from all participants. PBMC were separated using Ficoll-Paque PLUS (GE Health) density gradient method and were cryopreserved in R10 medium (RPMI-1640 (Gibco)), containing 10% heat-inactivated Foetal Bovine Serum (FBS) (Cytiva) containing 100 U/mL Penicillin and 100 µg/mL Streptomycin (Gibco) supplemented with 10% DMSO (Sigma-Aldrich). PBMC then advanced to the immune assay. The immunomodulatory effects of the bark extracts was assessed under three conditions. First, cytokine levels in unstimulated PMBCs were used as a negative control to determine the basal cytokine release (Fig. 3, green bars). Second, the effects on cellular immunity was examined (Fig. 4, Orange bars). Here, PBMCs were activated with a cell stimulation cocktail of 50 ng/mL of PMA and 1 µg/mL of ionomycin (eBioscience). Third, the effects on the myeloid compartment was examined (Fig. 5, red bars). Here, PBMCs were activated with 10 ng/mL lipopolysaccharide (LPS) (Sigma-Aldrich). For all tests, 100,000 cells in 100 μL of R10 media were seeded into the wells of round-bottom 96-well culture plates. Unstimulated PBMCs were treated in triplicate with 10 µg/mL of cyclosporine A (CsA) (Sigma-Aldrich) or individually with the *T impetiginosa* bark extracts from water (100 µg/mL), methanol (32 µg/mL), ethyl acetate (45 µg/mL), chloroform (100 µg/mL) or hexane extracts (100 µg/mL). Quality control PBMC cultures were treated with 10 µg/mL of CsA (positive control), whilst other cells were left untreated as negative controls. Plates were incubated for 24 h at 37 °C in a 6.5% CO_2_ incubator. Following overnight incubation, plates were centrifuged, and the culture supernatants were collected for cytokine analysis. All tests were performed in triplicate using the cytometric bead array (BD Biosciences). Levels of interferon gamma (IFN-γ), interleukin IL-1β, IL2, IL-6, IL-8, IL-10, monocyte chemoattractant protein-1 (MCP-1) and tumour necrosis factor (TNF-α) were quantified according to the manufacturers’ instructions and performed on a custom LSRFortessa X20 (BD Biosciences). Cytokine concentrations (all defined as pg/mL) were calculated based on the sample fluorescence intensity (MFI) compared to cytokine standard curves. The standard curve for IFN-γ could not be determined, thus this cytokine is reported as MFI. All tests were performed in triplicate and are expressed as mean ± standard deviation (SD). BD FCAP Array software version 3.0 was used for data analysis.

### Statistical analysis

Data is expressed as the mean ± SD of three independent experiments, each with internal triplicate. A one-way ANOVA with Dunnett’s multiple comparisons test an adjusted p-value was used to calculate statistical significance between treated groups. Bar graphs, ANOVA tables and statistics were generated using GraphPad Prism version 8.4.3 (GraphPad Software).

### Use of human participants

All protocols were carried out in accordance with guidelines and regulations of Scientific Reports and JCU (HREC) under (H7010). All methods and human participants involved in the study were approved by the JCU HREC under (H7010). Informed consent was obtained from all participants in the study under JCU HREC (H7010).

## Results

**Figure 1:**
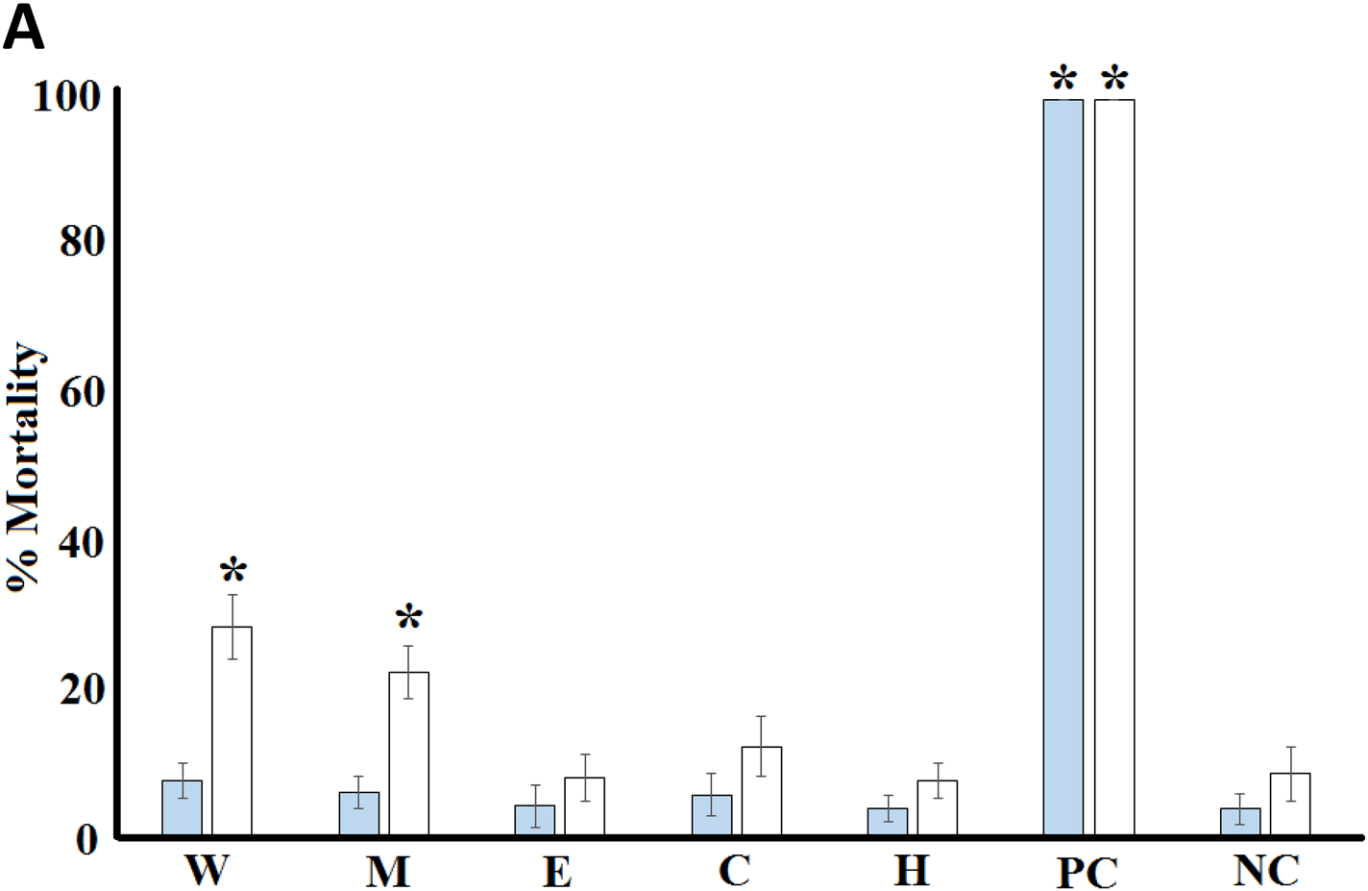
The toxicity of the *Handroanthus impetiginosus* (Mart. ex DC.) Mattos (Bignoniaceae) bark extracts. Potassium dichromate control (1000 µg/mL) and seawater (negative control) were included as controls. Shaded bars represent the mortality induced by the *Handroanthus impetiginosus* (Mart. ex DC.) Mattos (Bignoniaceae) extracts following 24 h exposure; open bars represent the mortality induced by the *Handroanthus impetiginosus* (Mart. ex DC.) Mattos (Bignoniaceae) extracts following 48 h exposure. All bioassays were performed three times in triplicate (n = 9) and are expressed as mean ± SD. *M* methanolic extract, *W* aqueous extract, *E* ethyl acetate extract, *C* chloroform extract, *H* hexane extract, *PC* potassium dichromate control, *NC* seawater (negative control). *Indicates results that are significantly different than the untreated (seawater) control at the equivalent exposure time (p < 0.01).

Extraction of 1 g of dried *Handroanthus impetiginosus* (Mart. ex DC.) Mattos (Bignoniaceae) bark with various solvents yielded dried plant extracts of 51.3 mg (water extracts) to 79.3 mg (hexane extracts) (Table 1). Methanol and hexane gave the highest yields of dried material, whilst all other solvents extracts, lower masses. The dried extracts were resuspended in 10 mL of deionised water (containing 1% DMSO), producing extracts concentrations shown in Table 1. None of the extracts exhibited mortality induction significantly different to the untreated control (sea water) following 24 h exposure (Fig. 1A). As all extracts induced < 50% mortality at all concentrations screened, all were deemed to be nontoxic.

**Table 1:** Qualitative phytochemical screenings of *T. impetiginosa* bark extracts.

Extract	H_2_O	MeOH	ETAC	CHCl_3_	HEX
Mass of extract (mg)	51.3	76.1	63.8	65.5	79.3
Concentration of the extract (mg/mL)	5.13	7.61	6.38	6.55	7.93
Phenolic compounds	+++	+++	+	+	+
Water soluble phenols	+	++	−	−	−
Water insoluble phenols	−	+++	+++	+++	+++
Cardiac glycosides	+++	+++	−	−	−
Polysteroids	−	−	−	−	−
Saponins	+++	−	+++	+++	−
Triterpenoids	+++	+++	+++	+++	+
Flavonoids	−	+++	++	+	+
Free anthraquinones	−	−	−	−	−
Combined anthraquinones	−	−	−	−	−
Mayer reagent test (alkaloids)	+++	+++	+++	+++	+++
(alkaloids)	+++	+++	+++	+++	+++

*H*_*2*_*O* water extract, *MeOH* methanol extract, *ETAC* ethyl acetate extract, *CHCl*_*3*_ chloroform extract, *HEX* hexane extract.

+++ indicates a strong response; +  + indicates a moderate response; + indicates a minor response; − indicates no response in the assay.

Qualitative phytochemical studies (Table 1) showed that methanol extract produced the widest range of phytochemicals, with high levels of phenolic compounds, cardiac glycosides, triterpenoids, flavonoids and alkaloids. The water extract produced high levels of phenolics, cardiac glycosides, saponins, triterpenoids and alkaloids. Ethyl acetate extract produced high levels of water-insoluble phenols, saponins, triterpenoids and alkaloids, as well as moderate levels of flavonoids and low levels of phenolic compounds. Similarly, the chloroform extract produced high levels of saponins, triterpenoids, alkaloids, as well as low levels of phenolic compounds and flavonoids. Hexane extract produced the narrowest range of phytochemicals, with water-insoluble phenols and alkaloids present in high abundance. Low levels of phenolic compounds, triterpenoids and flavonoids were also detected.

**Figure 2:**
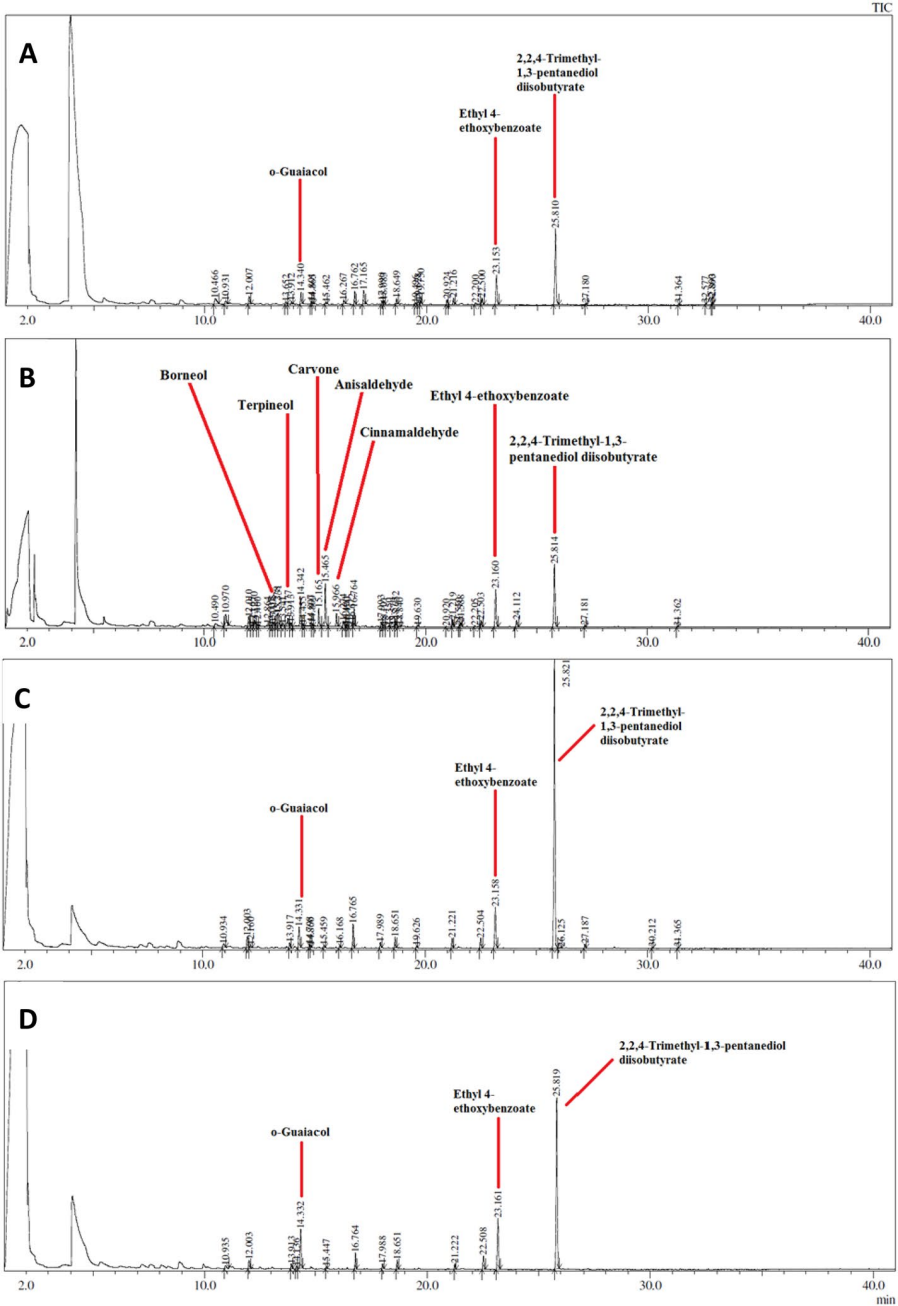
GC–MS headspace analysis of 0 µL injections of (**A**) aqueous; (**B**) methanolic; (**C**) ethyl acetrate and; (**D**) hexane *Handroanthus impetiginosus* (Mart. ex DC.) Mattos (Bignoniaceae) bark extracts with the major components (based on peak area) indicated.

Sixteen mass signals were detected in the ethyl acetate extracts chromatogram. Again, 2,2,4-trimethyl-1,3-pentanediol diisobutyrate was detected, although in substantially higher relative abundances compared to the other extracts (Fig. 2, Table 2). The ethyl acetate chromatogram also showed major peaks at 23.1 and 14.3 min. These peaks were putatively identified as ethyl 4-ethoxybenzoate and *o*-guaiacol, respectively. Notably, ethyl 4-ethoxybenzoate was detected in both aqueous and methanolic extracts (Fig. 2A,B), while *o*-guaiacol was only present in the methanol and ethyl acetate bark extracts (Fig. 2B,C). Multiple other peaks were also noted in the ethyl acetate extracts, with many of these corresponding to peaks in the aqueous and methanolic extracts. Interestingly, several novel peaks were identified in the ethyl acetate extracts that were not present in the methanolic and aqueous extracts. A total of 10 peaks were identified in the hexane extract (Fig. 2D). As for the water, methanol and ethyl acetate extracts, 2,2,4-trimethyl-1,3-pentanediol diisobutyrate was the most prominent peak, eluting at approximately 25 min. Ethyl 4-ethoxybenzoate and *o*-guaiacol were also detected as major components. However, *o*-guaiacol was detected in higher levels than in the polar and mid-polarity extracts (Table 2). Other overlapping peaks were also evident throughout the chromatogram, with a broad range of retention times between 10.9 and 25.8 min. Many of these peaks corresponded to peaks in the ethyl acetate extracts and higher polarity extracts at similar retention times. The hexane bark extracts GC–MS chromatogram was substantially less complex than the other bark extracts (Fig. 2D). Indeed, only seven peaks were evident in the hexane extracts chromatogram. Again, 2,2,4-trimethyl-1,3-pentanediol diisobutyrate was the major component (eluting at approximately 25 min). In addition, a further major peak was evident in the hexane extracts at approximately 23.1 min. A comparison to the phytochemical database putatively identified this compound as ethyl 4-ethoxybenzoate (Table 2). Both compounds were also present in the water, methanol, ethyl acetate and chloroform extracts. However, the 2,2,4-trimethyl-1,3-pentanediol diisobutyrate was present in substantially higher relative abundance in the hexane extracts (Fig. 2D, Table 2). Numerous overlapping peaks were also evident throughout the chromatogram, many at retention times corresponding to peaks in the chloroform extracts, indicating that hexane and chloroform extracted similar components.

**Table 2:** Qualitative GC–MS headspace analysis of the T. impetiginosa bark extracts, elucidation of empirical formulas and putative identification of each compound.

RT	MW	Empirical formula	Putative identification	Area%				
				M	W	E	C	H
10.49	120.06	C_8_ H_8_ O	*m*-Tolualdehyde	1.68	4.53			
12.27	138	C_8_ H_10_ O_2_	*o*-Dimethoxybenzene	2.21				
12.4	152	C_10_ H_16_ O	(+)-2-Bornanone	0.72				
12.864	237	C_13_ H_19_ NO_3_	3-Nitro-1-phenylheptan-1-ol	1.41				
13.105	154	C_10_ H_18_ O	l-Borneol	1.26				
13.541	152	C_8_ H_8_ O_3_	Methyl salicylate	1.09	0.99			
13.777	154	C_10_ H_18_ O	*α*-Terpineol	2.41	1.15			
13.913	212	C_15_ H_32_	Pentadecane	0.94		1.07	1.27	1.26
14.342	124	FC_7_ H_8_ O_2_	*o*-Guaiacol	8.24	4.17	4.41	10.96	0.77
15.165	150	C_10_ H_14_ O	d-Carvone	4.71				
15.465	136	C_8_ H_8_ O_2_	*p*-Anisaldehyde	9.63	0.63	0.49	0.52	
15.966	132	C_9_ H_8_ O	*trans*-Cinnamaldehyde	2.93				
16.274	130	C_8_ H_10_ O_2_	*p*-Anisyl alcohol	1.53	1.55	0.52		
16.4	188	C_10_ H_20_ O_3_	Butyl 2-butoxyacetate	0.38				
16.672	150	C_10_ H_14_ O	Thymol	0.84				
17.993	286	C_16_ H_30_ O_4_	2,2,4-Trimethyl-1,3-pentanediol diisobutyrate	0.95	1.28	1.05	1.19	1.37
18.652	216	C_12_ H_24_ O_3_	Propanoic acid, 2-methyl-, 3-hydroxy-2,2,4- trimethylpentyl ester	1.58	1.9	1.79	2.16	2.51
19.63	226	C_14_ H_26_ O_2_	2,4,7,9-Tetramethyl-5-decyn-4,7-diol	0.2	1.24	0.34		
20.92	236	C_15_ H_24_ O_2_	2,6-di-tert-butyl-4-hydroxy-4-methylcyclohexa- 2,5-dien-1-one	0.11	1.85	1.89	1.4	
21.51	172	C_11_ H_24_ O	1-Undecanol	0.2				
21.588	166	C_9_ H_10_ O_3_	Methylvanillin	0.94				
22.503	206	C_14_ H_22_ O	3,5-Di-tert-butylphenol	1.53	2.86	2.17	4.03	0.73
23.16	194	C_11_ H14 O_3_	Ethyl 4-ethoxybenzoate	9.79	13.56	9.91	16.4	14.83
24.112	208	C_12_ H_16_ O_3_	Elemicin	1.81				
25.814	286	C_16_ H_30_ O_4_	2,2,4-Trimethyl-1,3-pentanediol diisobutyrate	16.69	36	65.55	53.72	72.97
31.362	278	C_16_ H_22_ O_4_	Diisobutyl phthalate	0.1	0.27	0.12		
32.58	278	C_16_ H_22_ O_4_	Dibutyl phthalate		0.2			

*RT* retention time, *MW* mass weight, *M* methanol extract, *W* water extract, *E* ethyl acetate extract, *C* chloroform extract, *H* hexane extract.

Optimised non-targeted GC–MS headspace analysis (GC–MS) was used to examine the *Handroanthus impetiginosus* (Mart. ex DC.) Mattos (Bignoniaceae) bark extracts to identify volatile low polarity compounds. A total of 15 peaks were detected in the aqueous *Handroanthus impetiginosus* (Mart. ex DC.) Mattos (Bignoniaceae) bark extracts (Fig. 2A), with the peak eluting at 25.8 min being the most prominent. Comparisons with a phytochemical library putatively identified this peak as the 2,2,4-trimethyl-1,3-pentanediol diisobutyrate. Other overlapping peaks were also evident throughout the chromatogram, with a broad range of retention times between 10.4 and 32.8 min. For the water extracted compounds, there were numerous peaks throughout the chromatogram identifying many compounds of widely varying polarity. The methanolic bark extracts GC–MS chromatogram (Fig. 2B) was more complex than the aqueous extracts chromatogram (Fig. 2C). Indeed, a total of 41 peaks were detected in this chromatogram, with major peaks at approximately 12.4, 13.1, 13.7, 15.1, 15.4, 15.9, 23.1, and 25.8 min. Similar to the aqueous extracts, 2,2,4-trimethyl-1,3-pentanediol diisobutyrate (eluting approximately at 25 min) was the major component. There were overlapping peaks in the chromatogram, many at retention times consistent to peaks in the aqueous extracts (Fig. 2A,B, Table 2). This indicates that methanol and water extracted many similar components, although many of the lower polarity compounds were more effectively extracted using methanol.

**Figure 3:**
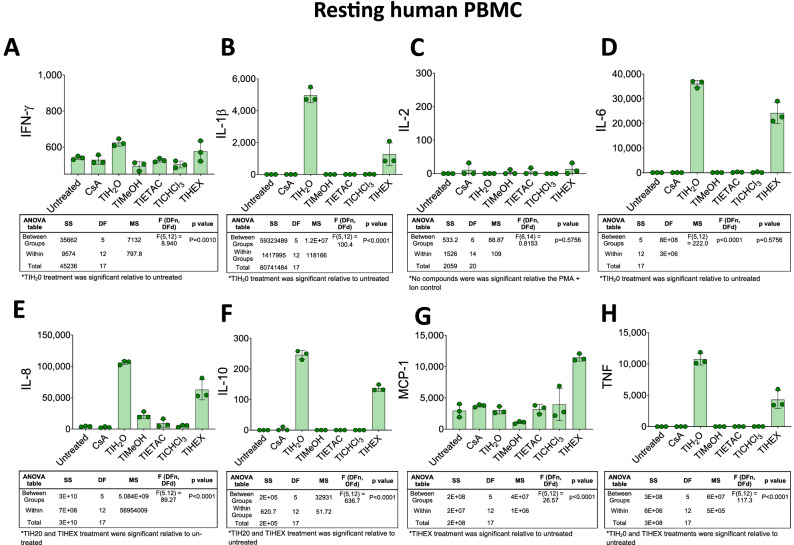
*Handroanthus impetiginosus* (Mart. ex DC.) Mattos (Bignoniaceae) bark-derived compounds can induce cytokine release at rest. PBMC were combined with TIH_2_O aqueous extracts, TIMeOH methanolic extracts, TIETAC ethyl acetate extracts, TICHCl_3_ chloroform extracts and TIHEX hexane extracts for 24 h. Individual cytokine levels of (**A**) IFN-γ, (**B**) IL-1β, (**C**) IL-2, (**D**) IL-6, (**E**) IL-8, (**F**) IL-10, (**G**) MCP-1 and (**H**) TNF were quantified by CBA as detailed in the “Material and methods” section. The data is the mean of three replicates ± SD. Associated ANOVA Tables are presented beneath the line graphs and significant or nonsignificant (*) results are shown beneath the Tables.

The *Handroanthus impetiginosus* (Mart. ex DC.) Mattos (Bignoniaceae) bark chemical extracts were next examined for their ability to modulate human immune cell function using methods previously described^25^. Briefly, CsA was used as a positive control for immune suppression. This drug blocks immune cell activation pathways and cytokine release^26^. For baseline immune measurement, PBMC supernatant was first screened at rest with five compounds (Fig. 3). Most extracts elicited no cytokine/chemokine release except for TIH_2_0 which significantly stimulated IFN-γ (Fig. 3A), IL-1β (Fig. 3B), IL-2 (Fig. 3C), IL-6 (Fig. 3D), IL-8 (Fig. 3C), IL-10 (Fig. 3F), MCP-1 (Fig. 3G) and TNF-α (Fig. 3H) release. TIHEX significantly stimulated IL-1β, IL-6, IL-8, IL10, MCP-1 and TNF-α release.

**Figure 4:**
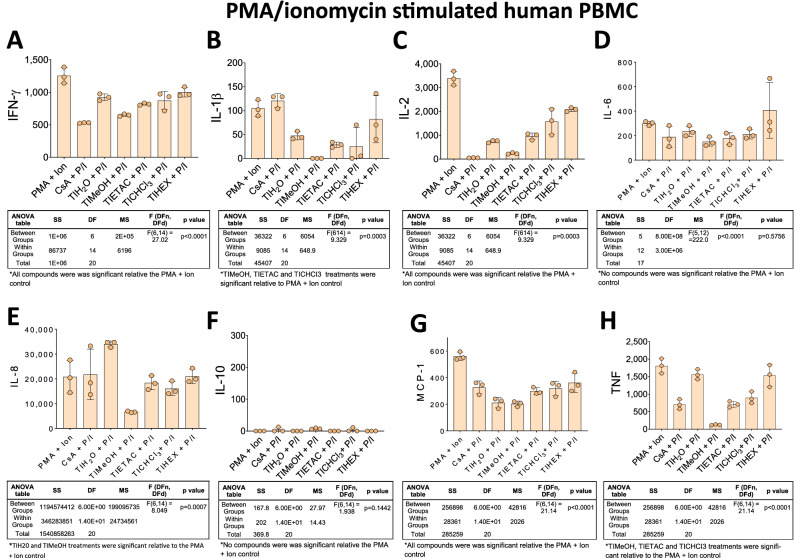
*Handroanthus impetiginosus* (Mart. ex DC.) Mattos (Bignoniaceae) bark-derived compounds can suppress cytokine release of PMA/Ionomycin-activated PBMC. PBMC were combined with TIH_2_O aqueous extracts, TIMeOH methanolic extracts, TIETAC ethyl acetate extracts, TICHCl_3_ chloroform extracts and TIHEX hexane extracts for 24 h. Individual cytokine levels of (**A**) IFN-γ, (**B**) IL-1β, (**C**) IL-2, (**D**) IL-6, (**E**) IL-8, (**F**) IL-10, (**G**) MCP-1 and (**H**) TNF were quantified by CBA as detailed in the “Material and methods” section. The data is the mean of three replicates ± SD. Associated ANOVA Tables are presented beneath the line graphs and significant or nonsignificant (*) results are shown beneath the Tables.

Next, we determine the suppressive activity of the extract compounds when PBMC were stimulated with PMA/ionomycin (Fig. 4). Interestingly, all compounds significantly suppressed inflammatory cytokines with different patterns and different potencies. The general suppressive potency of the extracts from TIMeOH < TIETAC < TIH_2_O < TICHCI_3_ < TIHEX. Notably, TIMeOH, suppressed 100% of IL-1β (Fig. 4B), 94% of TNF-α (Fig. 4H) and of 78% of IL-2 (Fig. 4B). All compounds could suppress IL-2 release to varying degrees. TIMeOH, TIETAC and TIHHCl_3_ compounds extracts could mildly suppress IL-6. No IL-10 was detected in any condition, and TIMeOH, TIETAC and TIH2O extract compounds could suppress TNF-α release to varying degrees. All extract compounds could suppress the chemokine MCP-1 to varying degrees with TIMeOH being the most potent. TIMeOH, TIETAC and TIHHCl_3_ compound extracts could suppress the key inflammatory marker TNF-α.

**Figure 5:**
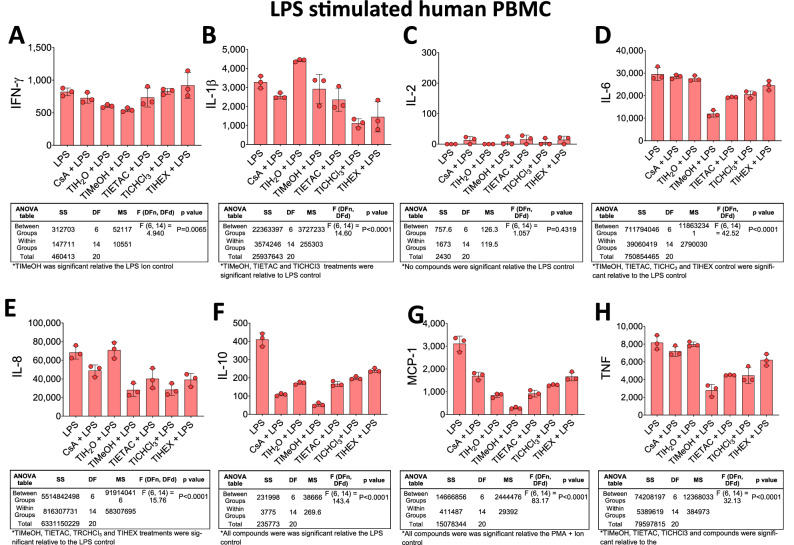
*Handroanthus impetiginosus* (Mart. ex DC.) Mattos (Bignoniaceae) bark-derived compounds can suppress cytokine release of LPS-activated PBMC. PBMC were combined with TIH_2_O aqueous extracts, TIMeOH methanolic extracts, TIETAC ethyl acetate extracts, TICHCl_3_ chloroform extracts and TIHEX hexane extracts for 24 h. Individual cytokine levels of (**A**) IFN-γ, (**B**) IL-1β, (**C**) IL-2, (**D**) IL-6, (**E**) IL-8, (**F**) IL-10, (**G**) MCP-1 and (**H**) TNF were quantified by CBA as detailed in the Material and Methods. The data is the mean of three replicates ± SD. Associated ANOVA Tables are presented beneath the line graphs and significant or nonsignificant (*) results are shown beneath the Tables.

Finally, we determined suppression when PBMC were stimulated with LPS (Fig. 5). Compound-induced immune modulation exhibited a different pattern to PMA, indicating a combination of bioactive compounds modulating different immune pathways. The potency was not as clear as PMA suppression, but TIMeOH was again the most suppressive agent against 5-of-6 cytokine/chemokines (Fig. 5A–D). The most significant suppression was against IL-10 at 87% and MCP-1 91% reduced release (Fig. 5F). Virtually all extracts showed suppression against LPS-induced IL-6, IL8, IL-10, MCP-1 and TNF-α production. Using LPS-stimulation, MCP-1 was more strongly suppressed compared with PMA-stimulation.

## Discussion

*Handroanthus impetiginosus* (Mart. ex DC.) Mattos (Bignoniaceae) bark powder has been used for millennia in treating inflammatory disorders. To date, the compounds from this powder have not been fully examined. Here, bark extracts were investigated for their ability to suppress human immune cells directly ex vivo.

*Handroanthus impetiginosus* (Mart. ex DC.) Mattos (Bignoniaceae) immunology studies have examined rodent models or cell lines. One examined the therapeutic effect of ground *Handroanthus impetiginosus* (Mart. ex DC.) Mattos (Bignoniaceae) bark in the drinking water in mouse using the DSS model of colitis^27^. From splenocytes, they determined the powder was nontoxic. Using flow cytometry and ICS, a mild-to-moderate increase in MHC-II and CD86 was observed. A minor-to-mild increase in anti-inflammatory and a decrease in pro-inflammatory cytokines was also observed. An improvement of weight and disease activity score was observed in DSS treated mice. In a human study using *Handroanthus impetiginosus* (Mart. ex DC.) Mattos (Bignoniaceae) bark power solubilised in EtOH, a nontoxic effect was also observed^28^. Using ground *Handroanthus impetiginosus* (Mart. ex DC.) Mattos (Bignoniaceae) bark, T cell proliferation was observed to increase in a concavalin A (ConA)/MTT assay which measures cell division. Post PHA stimulation, no changes in IL-2 or TNF-α were observed in whole blood during dosing using flow cytometry or ICS. This conflicts with our study where PHA-stimulated PBMC-stimulated produced clear secretion of 5-of-6 quantified cytokines. Of note, ConA is toxic to cells^28^ and MTT is a marker of cell metabolism and not specifically cell proliferation. Additional differences between results are *Tabebuia* species and the short 4 h ICS incubation. Our CBA assays quantified cytokines in supernatant after 24 h of stimulation which would likely increase sensitivity. Adding to mounting suppressive data of *Handroanthus impetiginosus* (Mart. ex DC.) Mattos (Bignoniaceae) bark, a recent study suggests that dissolving *Handroanthus impetiginosus* (Mart. ex DC.) Mattos (Bignoniaceae) bark powder in water could prevent DSS-induced colitis in mice^29^.

As shown in Fig. 2, GC–MS headspace analysis determined that each extraction method produced multiple compounds with likely, multiple cell activities. Across, resting state, PMA and ionomycin-stimulation, and LPS-stimulation conditions, all extracts significantly differed in activating/suppressing cytokine patterns and potencies. For example, at rest, PBMC could induce large amounts of IFN-γ, IL-1β, IL-8, IL-10 and TNF-α when combined with TIH2O aqueous extracts. The same was observed for the TIHEX hexane extracts with a similar profile and an added increase in MCP-1. Identifying these molecules could help drug development for use against infectious diseases and cancer (immunotherapy).

Interestingly, the immune-stimulating properties within the TIH_2_O aqueous extracts and TIHEX hexane extracts could be overridden by counter compounds in the bark that induce suppression. An example is TIH_2_O aqueous extracts which produced a 4966% increase in IL-1β at rest but was wholly suppressed in PMA-activated cells. The same inversion could be said for IL-10 and, in LPS-activated PBMC, IFN-γ and IL-10. Like the LPS observations, were compounds in TIHEX hexane extracts that modulated IL-1β, IL-10, IL-8, MCP-1 and TNF-α.

Some important compounds were determined in TIMeOH methanolic extracts, which could switch off the powerful cytokines IL-2 and TNF-α and significantly reduce IFN-γ and IL-6, all involved in cytokine storm. In the myeloid compartment, compounds from TICHCl3 chloroform extracts were highly effective at suppressing IL-1β and compound/s in TIMeOH methanolic extracts were highly effective at suppressing IL-6. Almost all the extracts could significantly supress IL-8, IL-10, MCP-1 and TNF-α, with the most potent compound/s within the TIMeOH (methanolic extracts). Notably, some compounds even outperformed our CsA control, including TIMeOH (methanolic extracts) (IL-1β, IL-8, MCP-1 and TNF-α) and TIH_2_O (IL-1β and MCP-1). While PMA-induced and LPS-induced cytokines can be suppressed by compounds in *Handroanthus impetiginosus* (Mart. ex DC.) Mattos (Bignoniaceae)bark powder, it is likely complex and additive chemical and biological circuit.

The results of this study showed that several of the *Handroanthus impetiginosus* (Mart. ex DC.) Mattos (Bignoniaceae) bark extracts were potent inhibitors of pro-inflammatory cytokine secretion. The secretion of IL-1β and TNF-α were upregulated in virtually all inflammatory conditions. TNF-α activates a cascade of inflammatory events by stimulating other cytokines including IL-1β, IL-6, I-L8 and granulocyte macrophage-colony stimulating factor (GM-CSF)^30^. TNF-α also regulates intracellular and vascular adhesion molecules (ICAM and VCAM)^31^. Several TNF-α inhibitors have been developed to reduce the production of IL-1β^32^ and have led to the implementation of anti-TNF-α therapy. Today, five anti-TNF-α drugs are available clinically (infliximab, adalimumab, golimumab, certolizumab and etanercept). However, these drugs have side effects and are expensive. Not all patients are responsive to anti-TNF-α antibody therapy^33^ and patients are at high risk of pathogen infections such as tuberculosis, non-tuberculosis mycobacteria and hepatitis C^34–37^.

The phytochemistry of the *Handroanthus impetiginosus (Mart. ex DC.) Mattos* (*Bignoniaceae*) bark extracts were examined to highlight which compounds may contribute to the cytokine-based modulatory effects reported in our study. As several previous studies have reported that some monoterpenoids have potent anti-inflammatory and immunomodulatory activity, we chose to use GC–MS analysis to detect volatile, lower polarity compounds. A notable feature of this analysis was the relatively low levels and low diversity of monoterpenoids was seen. However, several monoterpenoids were identified in the *Handroanthus impetiginosus* (Mart. ex DC.) Mattos (Bignoniaceae*)* bark extracts, including 2-bornanone, borneol, terpineol and carvone. Monoterpenes have been reported to suppress NF-Кβ signalling^38^. Despite the low relative abundance of terpenoids in *Handroanthus impetiginosus* (Mart. ex DC.) Mattos (Bignoniaceae) bark extracts, the presence of monoterpenoids 2-bornanone, borneol, terpineol and carvone are particularly interesting. Carvone has been reported to dissipate the transmembrane pH gradient and cell potential, thus disrupting general metabolic function^39^. This is due to their small hydrophobic properties, which allow them to enter and alter the flow and homeostasis of the cytosolic membranes, causing a change in structure and function. Consequently, this blocks several pathways in the cell and may contribute to the modulation of cytokine secretion. Similarly, 2-bornanone (identified in water extracts) has anti-inflammatory effects and is often used to treat rheumatic pain^40^. These findings suggest that borneol and bornanone could have an important role in the anti-inflammatory drug development.

**Figure 6:**
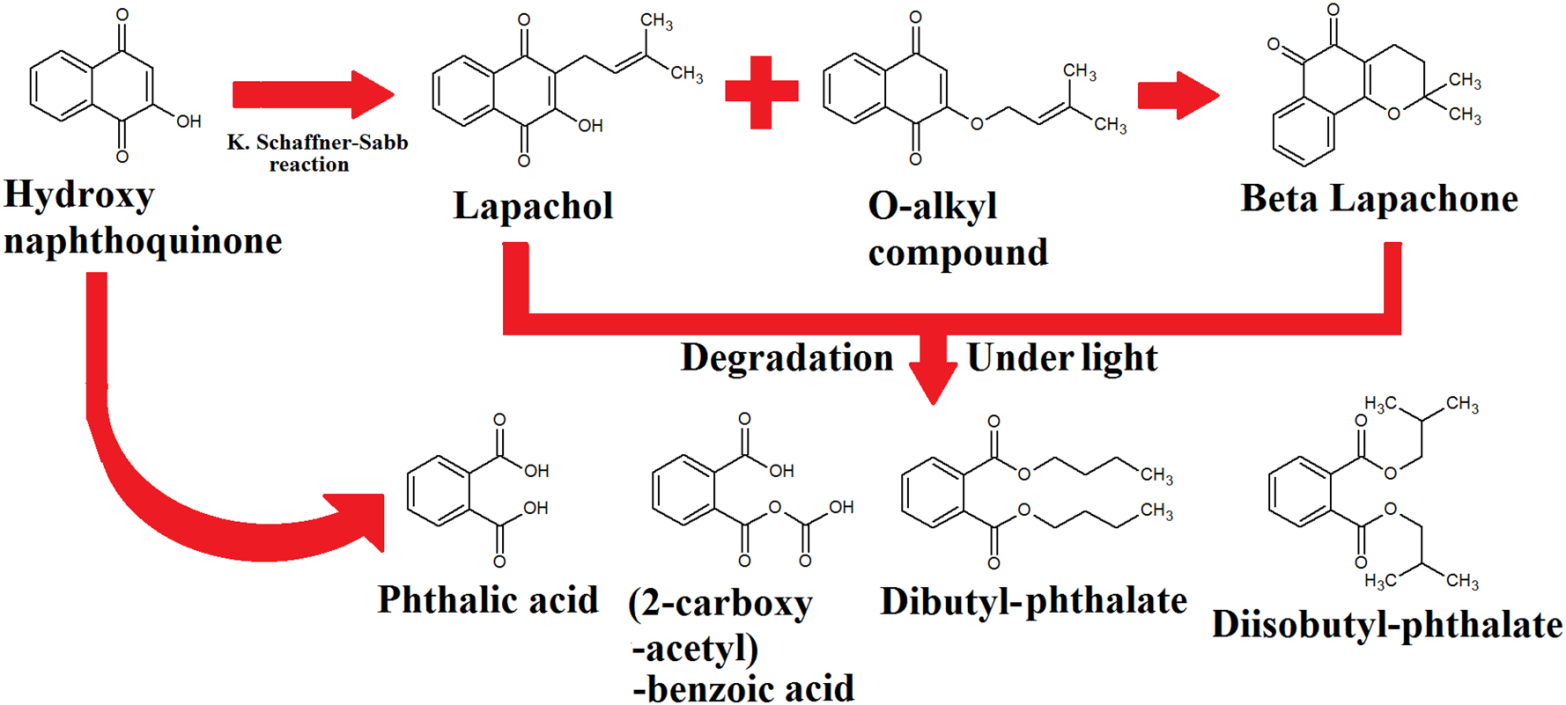
Lapachol, β-lapachone and the phthalates can all be synthesised directly from naphthoquinones. Under light conditions, the phthalates may also arise from lapachol and β-lapachone degradation.

However, it is likely that other compound classes may also contribute to the bioactivities reported in our study. Several benzene derivatives including 4-anysaldehyde, cinnamaldehyde, ethyl 4-ethoxybenzoate and o-guaiacol were also putatively identified in this study. This correlated with previous studies undertaken on *Handroanthus impetiginosus* (Mart. ex DC.) Mattos (Bignoniaceae) bark volatile/low polarity compounds by GC–MS analysis^41,42^. The detection of dibutyl phthalate and diisobutyl phthalate were particularly interesting. These compounds may be synthesised from naphthoquinones (Fig. 6) and are noteworthy due to their immunomodulatory activity and inflammatory properties^43,44^. Alternatively, dibutyl phthalate and diisobutyl phthalate may also be produced as by-products of the degradation of lapachol and lapachone, which are also synthesised from naphthoquinone precursors^45^. Extensive degradation of lapachol and lapachone to the phthalates occurs spontaneously under direct visible light^46^. As both lapachol and lapachone are abundant in *Handroanthus impetiginosus* (Mart. ex DC.) Mattos (Bignoniaceae) bark extracts and have been linked to the anti-inflammatory and anticancer activities properties of this species^8,14,15^, it is possible that the dibutyl phthalate and diisobutyl phthalate identified in our study may be indicative of the presence and degradation of lapachol and lapachone. Other relevant secondary metabolites detected in the extracts were triterpenoids and saponins. Saponins can disrupt membranes and have anti-inflammatory, anticancer and immune-stimulating properties^47^.

These results highlight the potential therapeutic use of *Handroanthus impetiginosus* (Mart. ex DC.) Mattos (Bignoniaceae) bark against infection, cancer and autoimmunity. The extracts are nontoxic, indicating their suitability for therapeutic usage. However, further work is needed to stringently identify bioactive molecules, synthesise them in the laboratory, preclinical testing and liaising with the compounds founders to put in place a shared agreement. Indeed, studies such as this verify traditional knowledge and help to safeguard its importance.
